# 

**Published:** 1916-02

**Authors:** 


### CONTENTS.

ORIGINAL COMMUNICATIONS—

"Lest We Forget." By J. Clarence Johnson, M. D., Atlanta, Ga. ........ 517

Sickness Insurance. A Preventive of the Neglect of Sick Individuals, of Charity Practice and of Unpaid Doctor's Bills. By Ira W. Bullard, M. D., Opelika, Ala. ........ 527

An Interesting Case of Inherited Syphilis. By A. H. Lindorme, M. D., Major Medical Corps National Guard, Georgia ........ 534

Regular Meeting of the Fulton County Medical Society, January 20. 1916. Reported by Allen H. Bunce, M. D. ........ 538

Regular Meeting of the Fulton County Medical Society, February 3, 1916. Reported by Allen H. Bunce, M. D. ........ 542

Fulton County Medical Society. Regular Meeting February 17, 1916 ........ 546

EDITORIAL ........ 551

SELECTIONS AND ABSTRACTS ........ 554

BOOK REVIEWS ........ 562

